# Actions at Law

**Published:** 1901-02-16

**Authors:** 


### The Hospital

A Journal of

The Medical Sciences and Hospital Administration.

Vol. XXIX.—No. 751.] Edited by Sir Henry Burdett, K.C.B., and Solomon C. Smith, M.D., M.R.C.P. February 16, 1901.

### Actions at Law.

The first edition of Johnson's Dictionary, containing a few definitions afterwards modified by the author, is not in these days easy of access; and we have therefore not been able to verify the current tradition, which in the days of Wilkes and Junius was in all probability well founded, that he explained "patriotism" to be "the last refuge of a scoundrel." Were he now living, and a co-worker with Dr. Murray and Mr. Henry Bradley, he would probably remove the explanation to another page of the volume, and would make it applicable to an "action for libel." The late Colonel Charteris, who, according to Arbuthnot's well-known epitaph, lived throughout the whole of a long life in the daily pursuit of every human vice, excepting prodigality and hypocrisy, was wont to say that he would readily give ten thousand pounds for a good character, because he could make a hundred thousand out of the possession. It must, we think, be a somewhat similar calculation which induces some plaintiffs to appeal to the bandaged eyes of justice, and even induces them sometimes to exhibit their own morality, integrity, and truthfulness in the witness-box, under the fierce light thrown upon these qualities by an expert cross-examiner.

Members of our profession are perhaps more exposed than any other class of persons to actions of this kind, generally founded upon the most frivolous pretexts, and often brought by persons in circumstances so needy as to be satisfied with a very small amount of so-called "compensation." It has too often happened, moreover, that medical men have paid money to escape the certain cost and worry of a defence, and under the illusory hope of "settling," once for all, a claim to which their first sign of weakness has served to give an enduring vitality. In dealing with such cases, the two kindred societies, the Medical Defence Union and the Medical Protection Society, have done excellent service, and, by the mere fact of showing to intending plaintiffs and to speculative solicitors that they would be opposed by the purse and by the legal advisers of a united body, have been able to nip in the bud many ingenious endeavours at extortion. They might, we think, do even more if they would make an earnest attempt to carry the war into the enemy's country, and to surround the initiation of actions with difficulties which would present serious obstacles to unprincipled litigants and to their lawyers.

As the law now stands, anyone is able to bring an action for alleged damage against any person whom he may select, and is able to compel his victim to pay large sums of money, which he may possibly be very ill-able to afford, in mere defence against unprovoked aggression. In an action tried in the King's Bench Division of the High Court last week, the defendant, a clergyman, and presumably not a very rich man, had practically no choice but to retain Sir Edward Clarke as his leading counsel, and Sir Edward is a gentleman who deservedly obtains high fees. It was part of the plaintiff's case that he had been deprived of his means of living by the defendant's conduct, and that he had been forced to enlist in the Yeomanry and to proceed to South Africa; a statement quite sufficient to suggest the belief that nothing in the way of costs will ever be extracted from him. The jury found for the defendant; the effect of this finding being to show that the reverend gentleman had acted with perfect propriety, and to show this at a cost to him of at least several hundred pounds, which, to say the very least, might have been expended in a more agreeable and probably in a more useful manner. It would be easy to multiply similar instances, although in most of them smaller sums would be involved; and it is probably true that the medical profession, as a class, is more exposed to this sort of thing than any other, unless, indeed, it be journalists. The clergy are only occasional victims, and have not yet felt the necessity of combining for self-defence. Has not the time come at which the two Medical Societies to which we have referred might usefully seek to bring the whole subject under the consideration of the Legislature, and to obtain the passage of an enactment providing that a plaintiff should either give complete security to the defendant for all the legitimate costs of the defence, or else that he should satisfy some public official, such as the Attorney-General or the Public Prosecutor, that he had real ground of action. Such an enactment would, of course, be opposed by lawyers upon a plea of possible hardship to poor plaintiffs. All such considerations would be entirely met by the suggested appeal for permission to a public officer, and a grave scandal might without much difficulty be removed from our judicial proceedings. If the medical societies would move in the matter they would before long obtain the assistance of the Press, and, although abuses die hard, they may usually be killed in time.

